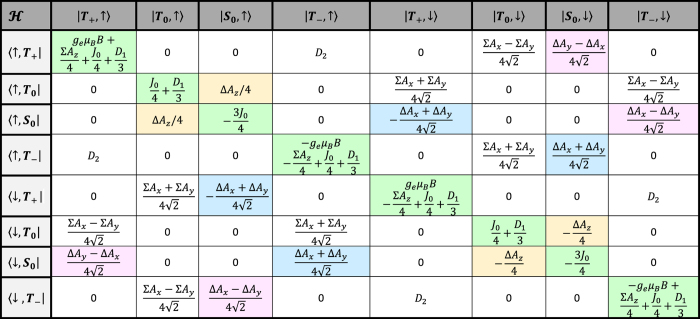# Vectorized magnetometer for space applications using electrical readout of atomic scale defects in silicon carbide

**DOI:** 10.1038/srep37077

**Published:** 2016-11-28

**Authors:** Corey J. Cochrane, Jordana Blacksberg, Mark A. Anders, Patrick M. Lenahan

**Affiliations:** 1 Jet Propulsion Laboratory, California Institute of Technology, Pasadena, CA 91109, USA; 2 The Pennsylvania State University, University Park, PA 16802, USA

## Abstract

Magnetometers are essential for scientific investigation of planetary bodies and are therefore ubiquitous on missions in space. Fluxgate and optically pumped atomic gas based magnetometers are typically flown because of their proven performance, reliability, and ability to adhere to the strict requirements associated with space missions. However, their complexity, size, and cost prevent their applicability in smaller missions involving cubesats. Conventional solid-state based magnetometers pose a viable solution, though many are prone to radiation damage and plagued with temperature instabilities. In this work, we report on the development of a new self-calibrating, solid-state based magnetometer which measures magnetic field induced changes in current within a SiC pn junction caused by the interaction of external magnetic fields with the atomic scale defects intrinsic to the semiconductor. Unlike heritage designs, the magnetometer does not require inductive sensing elements, high frequency radio, and/or optical circuitry and can be made significantly more compact and lightweight, thus enabling missions leveraging swarms of cubesats capable of science returns not possible with a single large-scale satellite. Additionally, the robustness of the SiC semiconductor allows for operation in extreme conditions such as the hot Venusian surface and the high radiation environment of the Jovian system.

The planetary science community has identified a strong need for scientific instruments which address crosscutting themes of planetary science: building new worlds, planetary habitats, and workings of the solar system^1^. Addressing aspects of all of these themes, magnetometers remotely probe the interiors of solar system bodies without the need to invasively penetrate the bodies being investigated. Magnetic field measurements have been used to better understand the internal workings of the planetary objects and have also been used in conjunction with simulations and models to shed insight into the predictive formation and evolution of the planets, satellites, and even the solar system. They also allow for a better understanding of planetary atmospheres and their interaction with the solar wind which influences climate and the ability to harbor life. Magnetic field measurements have also been useful for indirect detection of water, a requirement for life as we know it. For example, the magnetometer aboard the Galileo spacecraft that passed by Jupiter in 1996 acquired data that corroborated the idea that liquid water is present beneath the ice sheet of Europa, one of Jupiter’s moons. Specifically, computer modeling has shown that a planetary-scale conductive liquid ocean is necessary to explain the complicated, time-varying magnetic measurements of the Jovian environment^2^,^3^,^4^. More recently in 2005, the magnetometer on the Cassini spacecraft which will soon begin its grand finale tour of Saturn’s innermost rings, helped determine that the Saturn moon Enceladus is also likely to have a subsurface liquid ocean. By measuring the gyration frequency of the ionized gas ejected from the moon’s southern plumes in the presence of Saturn’s magnetic field, it was determined that the outgassing material is consistent with ionized water vapor^5^,^6^,^7^. These magnetometer-aided scientific discoveries are exciting because they suggest the existence of possible life sustaining environments beyond Earth. So much interest has been generated around these two moons that future missions are currently being planned to enable more detailed scientific investigations. A dedicated mission to Europa has recently been developed toward a proposed launch sometime in the 2020’s which would be equipped with a suite of magnetometers (both flux gate and optically pumped helium gas) called the Interior Characterization of Europa using Magnetometry (ICEMAG)^8^,^9^. The objective of ICEMAG is to characterize the complex magnetic environment of the Jupiter system at Europa at multiple frequencies, which will allow for a better understanding of the moon and potential life harboring environments beneath the ice. ICEMAG plans to use magnetic field measurements to determine the induction response at Europa, leading to constraints on the thickness and salinity of Europa’s ocean as well as the thickness of the ice shell. An additional ICEMAG goal is to use magnetic field measurements to probe the composition of molecular species picked up from Europa’s exosphere, as well as from any potential plumes that may exist, by detecting ion cyclotron waves at characteristic frequencies. As our ability to glean more and more information about planetary bodies grows along with sophisticated modeling of their magnetic environments, so does the pressing need for more aggressively miniaturized, high sensitive and robust magnetometers. This is the motivation for the work described herein.

## Previous Work

Even though an extensive set of magnetometers have been developed over the past few decades^10^,^11^, only a subset are suitable for measuring DC magnetic fields in space and around other planetary bodies^12^,^13^. Of this limited set, the most widely utilized in space missions are fluxgates and optically pumped atomic gas. Solid-state based magnetometers, such as Hall and magnetoresistive based sensors, have only recently been seriously considered for space missions due to the relative infancy of the technology and the uncertainty of performance in the harsh environments of space. However, they are gaining significant interest as the semiconductor industry is continually making large strides to improve material systems that are resilient to the highly varying temperatures and radiation environments typically encountered in space and other planetary systems.

Fluxgate magnetometers are most often used for near-zero DC magnetic field sensing in space applications where simplicity and cost are of the highest concern. A fluxgate magnetometer is composed of a ferromagnetic core wrapped with three sets of coils. The first set of coils is used to drive the core into and out of saturation, and the second set is used to sense the induced effect. Because the response of the ferromagnetic core is nonlinear, the induced signal will be rich in harmonics which can be used as a reference to null the external field with a third set of coils. Fluxgate magnetometers have flown on an extensive list of missions; some of those worth noting include the two Voyager missions on the Planetary Grand Tour encompassing Jupiter, Saturn, Uranus, Neptune, Pluto, now venturing into interstellar space, the Ulysses mission to study the Sun^14^, the Cassini mission to Saturn^15^, the MESSENGER mission to Mercury^16^, the BepiColombo Mercury Planetary Orbiter^17^, the MAVEN mission to Mars^18^, and the Juno mission to Jupiter which arrived on July 4^th^ 2016^19^. While not the most sensitive option for space bound magnetometers, fluxgate sensors are moderately stable and can exhibit high sensitivity 
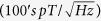
. Sigma-delta ADC technology with improved sampling resolution has been leveraged to enhance sensitivity of this technology^20^,^21^. Even though significant progress has been made to miniaturize these magnetometers^22^, the incorporation of a magnetically susceptible core wrapped with driving, sensing, and nulling coils for each axis will always limit the size and weight, and will never lead to a purely electrical device.

For applications where higher sensitivities and better stability are required, optically pumped He magnetometers are the most common choice. These magnetometers have flown on many missions including the Pioneer 10–11 missions to Jupiter and Saturn^23^, Ulysses^14^, Cassini^15^,^24^, SWARM^25^, and Juno^19^. They will also be flown on the upcoming NASA mission to Europa^8^. While being the most sensitive and stable magnetometers used in space, the optically pumped helium magnetometers are more complex as their scalar based design (can also be configured in vectorized mode) requires two sources of RF excitation, an optical pumping source, and an optical sensor. The first RF source excites the He atoms from their ground state 1^1^*S*_0_ to a metastable state 2^3^*S*_1_. This transition forces the electrons within the atoms from their singlet state (net spin angular momentum of *S* = 0, *m*_*s*_ = 0) to a triplet state (*S* = 1, *m*_*s*_ = −1, 0, +1) which makes the system electron paramagnetic resonance (EPR) active. Optical pumping of the metastable He gas using right-hand circularly polarized light with *λ* = 1083 *nm* provides a source of angular momentum *m*_*J*_ = −1 to depopulate the *m*_*s*_ = +1 state into the 2^3^*P*_0_ excited state which results in a system with net magnetization due to the remaining electrons in the *m*_*s*_ = −1 state. (Note that left-hand circularly polarized light with angular momentum of *m*_*J*_ = +1 could also be used to depopulate the *m*_*J*_ = −1 state.) After some time, the majority of the photons from the optical source will completely pass through the gas because absorption of the *m*_*s*_ = −1, 0 states is forbidden due to conservation of angular momentum. An optical detector on the opposite side of the He gas monitors the optical pumping efficiency and controls the frequency *v* of a second RF source which maintains the EPR condition of the electrons in the *m*_*s*_ = −1 state. The resonance condition is defined by *hv* = Δ*m*_*s *_*gμ*_*B*_*B*, where *h* is Planck’s constant, Δ*m*_*s*_ is the difference in spin angular momentum quantum numbers of the spin states, *g* is the electron’s g-factor, *μ*_*B*_ is the Bohr magneton, and *B* is the external magnetic field to be measured. When satisfied, the electron spins are able to flip which eventually equally populates the metastable states thereby destroying the magnetization. This allows for an increase in optical pumping efficiency to be measured by the optical detector. The frequency *v* is therefore a direct measure of the external magnetic field in which the He gas is immersed. (A more detailed analysis of this magnetometer can be found in refs 23 and 24). These magnetometers achieve the highest sensitivity and stability for space applications at the cost of using high frequency RF and optical circuitry. These components not only add cost, size, and complexity to the instrument, but they also require stable temperatures for operation.

Solid-state based magnetometers alternatively have very limited history in space. They fall into three main categories: Hall based sensors, magnetoresistance based sensors, and the new and emerging semiconductor defect magnetic resonance based approaches. Hall sensors function by measuring a voltage across an appropriately biased, precisely doped semiconductor. Hall sensors are not ideal for sensitive magnetic field measurements in space because they have a sensitivity proportional to magnetic field strength, making them ill-suited for near-zero field sensing. They are also prone to temperature drift as well as prone to radiation damage as most are typically made from silicon. However, SiC based Hall sensors have previously been developed for higher magnetic field sensing applications^26^. Magnetoresistance based devices usually involve magnetic field induced changes in resistance of complex layered ferromagnetic structures, some of which include ordinary (OMR), anisotropic (AMR), giant (GMR), and tunneling magnetoresistance (TMR). Although many forms of magnetoresistive sensors exist, only a few have been considered for space missions, those mainly being AMR^27^,^28^,^29^,^30^,^31^,^32^ and also TMR^33^. Though these sensors have shown much progress in the past few years (e.g. 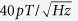
noise floor for AMR sensor^28^), there still remains reliability concerns with their overall stability, temperature stability, and radiation hardness. Additionally, even though these types of sensors can be made extremely small, because most of their responses are orientation dependent, multiple sensors are usually required for simultaneous measurement of three axes which can add complexity. The solid-state magnetic resonance based approaches, including EPR^34^, electrically detected magnetic resonance (EDMR)^35^,^36^,^37^ and optically detected magnetic resonance (ODMR)^38^,^39^,^40^,^41^,^42^,^43^, are based on detecting the resonances of unpaired electrons tied up in atomic scale defects within the semiconductor. Some of the more interesting work entail those that sense defects within the SiC material system as its wide bandgap allows for operation in high temperature and high radiation environments^34^,^37^,^40^,^41^,^42^,^43^. Even though a wide bandgap material is usually termed “rad-hard”, SiC still is prone to point defect creation in very high radiation environments. As a result, the tuned resonance condition of these magnetometers may be altered due to the creation of defects with g tensor components different than those intended to be sensed, thereby changing the anticipated response making the measurement unreliable. These approaches also rely on high frequency optical and radio circuitry which adds complexity, cost, and reliability concerns. As a result, a fully reliable solid-state magnetometer for space applications remains elusive to this day. The work outlined in this study is based on the latter two forms of solid-state magnetometers: it leverages atomic scale defects to sense magnetic fields; however, it measures the associated magneto response that allows for field measurements without any high frequency circuitry or optical components which simplifies the measurement.

## Magnetometer Description

The technique described herein involves the detection of magnetic fields using the recently demonstrated zero-field spin dependent recombination (ZFSDR) in SiC electronics^37^. The ZFSDR phenomenon provides these solid-state devices with magnetoresistive properties which makes them ideal for the development of miniaturized and purely electrical based magnetometers for near-zero magnetic field sensing (|*B*| < 10 *uT*). Similar to fluxgates, the proposed SiC magnetometer (SiCMag) is inexpensive, relatively simple to implement, and does not include high frequency RF or optical components which are sensitive to slight changes in temperature. Additionally, because only a single microelectronic device (with sensing area of less than 0.01 mm^2^) is required for simultaneous measurement of three magnetic axes, not only is the technology applicable to large scale missions, but it is also more adaptable to smaller missions which leverage nano- and picosats where fluxgate and optically pumped based designs are too large for implementation. (These smaller satellites are categorized as having a mass in the range 0.1 to 10 kg). Although this defect related magneto-response has been observed in Si based electronics^44^ and organic based semiconductors^45^,^46^,^47^, we focus on SiC based sensors, as its semiconductor properties are significantly more robust than its counterparts. Although the SDR phenomenon can be observed at temperatures as low as −260 °C, we do not plan on operating the magnetometer at this temperature. Like most magnetometers flown in space, the sensor will be housed in a small enclosure which will be space heated, likely no warmer than −120 °C (similar to the lower limit of some fluxgate magnetometers). Additionally, although the SiC semiconductor can operate reliably in temperatures to those encountered on the Venusian surface (≈460 °C), the magnetometer’s maximum operating temperature limit will likely be determined by the robustness of the supporting SiC electronics that will be eventually integrated. Current work in this area shows much promise as SiC ICs were demonstrated to reliably operate at 500 °C for more than 1000 hours^48^.

## SiC Sensor

At the heart of the proposed magnetometer lies a 4H-SiC pn junction. The junction’s sensitivity to magnetic fields arises from intrinsic, deep level, atomic scale defects that play a dominating role in SDR. When a device is biased to yield a recombination dominated current, semiconductor conduction electrons couple with electrons associated with deep level defects. These intermediate coupled pair states can be described by the singlet-triplet basis: the symmetric triplet states, *T*_+_ = |↑↑〉, *T*_0_ = (|↑↓〉 + |↓↑〉)/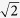, and *T*_−_ = |↓↓〉, each having spin angular momentum *S* = 1 with *m*_*s*_ = +1, 0, −1 respectively or the anti-symmetric singlet state, *S*_0_ = (|↑↓〉 + |↓↑〉)/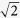 which has spin angular momentum *S* = 0 with *m*_*s*_ = 0. Because recombination conserves angular momentum, capture will only occur for singlet pairs whereas triplet pairs will dissociate after a given amount of time. The process is completed by subsequent electron-hole annihilation. (The order of electron/hole capture may of course be reversed).

In the absence of a magnetic field, the ratio of singlet to triplet pairings within the SiC will maintain a certain rate of recombination. However, the singlet to triplet population ratio can be altered by application of an external magnetic field which can be detected as a change in diode current. To understand how this occurs, first consider the Hamiltonian of the two electron spin system in the presence of neighboring magnetic nuclei immersed in a small external magnetic field directed in the *z* direction, i.e. 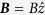,


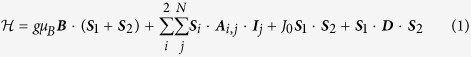


**Figure 1:**
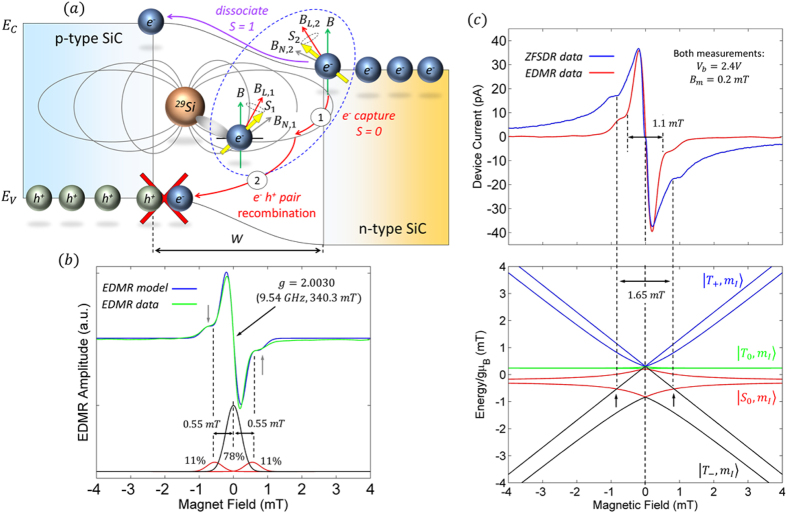
(**a**) Conceptualized illustration of SDR in a pn junction where singlet-triplet pairs form in the presence of a nuclear magnetic field *B*_*N*_ and a small external magnetic field *B*. The local field *B*_*L*_ experienced by each electron is the vector sum of both of these field components. Conduction electrons will couple with unpaired defect electrons for a finite amount of time forming either triplet or singlet states. Because the capture event, leading to eventual recombination, involves zero change in angular momentum, only singlet pairs will lead to recombination whereas triplet pairs dissociate. Because triplet pairs exist for a finite amount of time, radiative (magnetic resonance) or non-radiative transitions from triplet pairs to singlet pairs may occur which can increase the capture rate and thus the recombination rate. The latter occurs due to the mixing of states that results when the spin sites have slightly different local fields. See Table 1. (**b**) Comparison of the SDR response acquired via high-field EDMR (*B*_0_ = 340.3 *mT, v* = 9.54 *GHz, B*_*m*_ = 0.2 *mT*) and it’s corresponding model (see text for description). Note that the equally spaced satellite peaks, indicated by the vertical arrows spaced 1.1 *mT* apart, are consistent with a doublet involving hydrogen. (**c**, top) Comparison of the high-field EDMR and ZFSDR responses when biased with 2.4 *V*. (**c**, bottom). Energy levels of the spin Hamiltonian matrix, given by Table 1, evaluated over a small range of magnetic fields using hyperfine parameters obtained from the high-field EDMR model. Note that the hyperfine peaks illustrated in the ZFSDR response are precisely located at magnetic fields which correspond to crossing of singlet and triplet energy levels.

Here, *g* is a defect specific and (though not necessarily) an orientation dependent constant usually around 2 (assumed here to be the same for defect and charge carrier), *μ*_*B*_ is the Bohr magneton, ***S***_1_ and ***S***_2_ are the spin angular momentum operators of the two spins, ***I***_*j*_ are the nuclear spin angular moment operators for the *N* neighboring nuclei, ***A***_*i*,*j*_ the nuclear hyperfine parameters of electron *i* with nucleus *j, J*_0_ is the isotropic exchange constant, and ***D*** are the dipolar parameters. The magnetic isotopes in the SiC system are the host atoms of ^29^Si (*I* = 1/2, 4.7% abundant) and ^13^C (*I* = 1/2, 1.1% abundant) as well as the p-type dopant atom of ^27^Al (*I* = 5/2, 100% abundant). It is assumed for brevity that the hyperfine tensor is axially symmetric, and therefore has diagonalized elements *A*_*i*,*x*_, *A*_*i*,*y*_, and *A*_*i*,*z*_ for *i* = 1, 2. Consider the simplified case of a dangling bond deep level defect coupled to a ^29^Si atom within a forward biased SiC pn junction as illustrated in Fig. 1a. Although exaggerated in the figure, note that each electron will experience a slightly different local field ***B***_*L*,1_ and ***B***_*L*,2_ due to the differences in position of the two electrons surrounding the magnetic ^29^Si nucleus. The local field is simply the vector sum of the external field *B* and the nuclear field present at each of the spin sites, ***B***_*N*,1_ and ***B***_*N*,2_. In general, the nuclear fields are related to the hyperfine parameters by 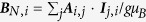. Table 1 illustrates the Hamiltonian matrix elements of the singlet triplet pair in the presence of a single nucleus (*N* = 1). Here, the ↑ and ↓ arrows indicate the spin orientation of the spin ½ nucleus. Σ*A*_*x*_, Σ*A*_*y*_, and Σ*A*_*z*_ are the sum of the hyperfine parameters at the two spin sites and Δ*A*_*x*_, Δ*A*_*y*_, and Δ*A*_*z*_ are their differences. Also, *D*_1_ = 3*D*_*z*_/2 and *D*_2_ = (*D*_*x*_ − *D*_*y*_) which naturally evolve from the axial symmetric dipolar matrix ***D*** with diagonal values of *D*_*x*_, *D*_*y*_, and *D*_*z*_. As illustrated in the table, the Δ*A*_*x*_ and Δ*A*_*y*_ hyperfine components mix the singlet states with the *T*_+_ and *T*_−_ states while the Δ*A*_*z*_ component mixes the singlet state with the *T*_0_ state.

The low-field hyperfine mixing alters the ratio of singlet to triplet pairings thereby allowing changes in recombination current to be measured for changes in external magnetic field. However, because the mixing coefficients are small, only small changes in current (up to a few percent) can be measured in the response. Therefore, frequency and phase synchronous detection is achieved via magnetic field modulation to enhance the sensitivity and signal-to-noise ratio of the measurement. This method restricts the measurement to the axis of modulation and allows for a vectorized measurement to be made. As a result, the responses are typically reported as derivatives which exhibit a sharp zero crossing that is approximately linear through zero magnetic field. As the magnetic field is increased, the mixing is suppressed by the Zeeman energy splitting. However, by exposing the spin system to electromagnetic (EM) radiation with energy equal to the splitting of the defect states, one is also able to measure a change in recombination current due to the modified populations of singlet and triplet states that occurs. This is more commonly known as EDMR. The EM radiation provides a source of angular momentum to the spin pair, thereby allowing the electrons to change state (or flip spin) if angular momentum is conserved and the resonance condition is satisfied, that being when 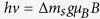. (Note that resonance also occurs at magnetic fields corresponding to *B* ± ***B***_*N*_ for a nucleus with *I* = 1/2 such as ^29^Si, ^13^C, or ^1^H) Essentially, triplet pairs are able to be converted to singlet pairs, thereby increasing the rate of recombination current and hence, depending on the circumstances, either an increase or decrease in resistance that can be detected electrically^49^. Figure 1b illustrates the SDR response, acquired via high-field EDMR (*B* = 340.3 *mT, v* = 9.54 *GHz*), of the device used in our study. The measurement reveals that the dominant defect is a silicon vacancy variant. (A previous EDMR study acquired from a nearly identical device directly linked the dominant defect spectrum to that of a negatively charged silicon vacancy^50^). With the crystalline c-axis of the SiC aligned with the external magnetic field, the acquired spectrum consists of a very strong dominating central line (96% of total spectrum), with two very small, almost negligible, sets of side peaks (1.5% and 0.5% of the central line) due to the 1.1% magnetic abundance of ^13^C. The strong electron-nuclear hyperfine side peaks that are apparent in the spectrum can be attributed to the, almost 100% magnetic abundant, spin *m*_*I*_ = ±1/2 ^1^*H* atom (1.1 *mT* splitting) which is present near the SiC/SiO_2_ interface. The defect is isotropic as the acquired spectra are nearly identical when the crystalline c-axis is rotated about three axes. Therefore, as illustrated in Fig. 1b, the response can simply be modeled with a strong dominating central line (78% of the total spectrum) with two side peaks (each contributing 11% to the defect spectrum) equally spaced 0.55 *mT* from the central line. Note that all three peaks in the model are generated from a Gaussian distribution with standard deviation *σ* = 0.05 *mT*. The peaks are summed and the derivative is taken to mimic the effect of magnetic field modulation. As illustrated from the figure, there is very good agreement between the model and the EDMR data.

Figure 1c illustrates the comparison of the high-field EDMR and the ZFSDR magnetoresistive responses acquired from the same device biased with the same potential. Even though the ZFSDR spectrum appears broader, it has a very similar zero-crossing slope compared to that of the high-field response. This makes the ZFSDR response an excellent technique for sensing near-zero magnetic fields (|*B*| < 0.1 *mT*). Remarkably, the hyperfine side peaks are retained in this simple measurement. By inspection of the eigenvalues of the Hamiltonian matrix evaluated at different magnetic fields using the isotropic defect hyperfine parameters obtained in the high-field EDMR model (*A*_1,(*x,y,z*)_*m*_*I*_/*gμ*_*B*_ = 0.55 *mT*) and that of a conduction electron located twice as far away (*A*_2,(*x,y,z*)_*m*_*I*_/*gμ*_*B*_ = 0.55/2^3^ *mT*), it is apparent from Fig. 1c that not only do the singlet and triplet pairs mix precisely at zero magnetic field (between the |*T*_+_, ↓〉 and |*S*_0_, ↑〉 states), but there is also a singlet-triplet intersystem crossing at *B* = ±0.825 *mT* which exactly corresponds to the location of the electron-nuclear hyperfine peaks observed in the ZFSDR spectrum. Because the hyperfine interactions are isotropic, it is precisely the mixing between the |*T*_−_, ↑〉 and |*S*_0_, ↓〉 states which allows the satellite peaks to be measured due to the preservation of the Δ*A*_*x*_ + Δ*A*_*y*_ matrix elements. (Note that the Δ*A*_*x*_ − Δ*A*_*y*_ and Δ*A*_*y*_ − Δ*A*_*x*_ elements cancel for isotropic defects). In general however, depending upon the strength and number of the hyperfine interactions, multiple satellite peaks may be detected in the ZFSDR response at fields relating to the hyperfine parameters themselves. This is clearly evident in more complicated defects as those reported in 4H-SiC BJTs^37^. Also, depending on the proximity of the two electron spins, dipolar and or exchange interactions may cause a split peak to present at zero magnetic field due to the singlet-triplet degeneracy removal that occurs from this interaction. These features are depicted in some of the spectra illustrated in latter sections of this report.

## Sensitivity

Because the magnetometer relies on a magnetic field modulation scheme, the sensitivity of the instrument can be defined by the zero crossing slope of the response and the noise retained in the bandwidth of interest of the modulation frequency used. (A review of magnetic field modulation as it relates to EDMR and intersystem crossing can be found in the work by Lee^51^). As we are only interested in the local derivative at the zero crossing center line, a Gaussian line shape can be assumed,


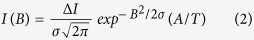


**Figure 2:**
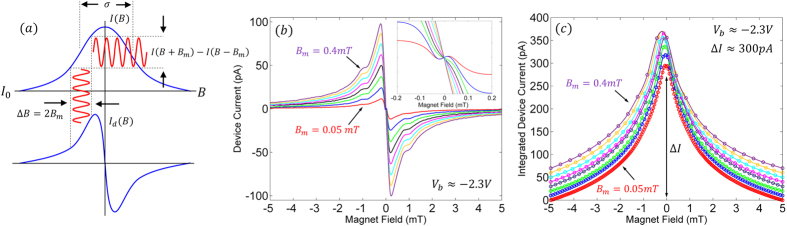
(**a**) Magnetic field modulation scheme illustrating the relationship between the line shape of the raw data (bottom) to the actual response (top). (**b**) Raw data obtained using a forward junction bias of 2.3 *V* at various modulation amplitudes. The inset of the figure illustrates the effect of increased applied junction bias which causes a split peak about 0mT to be observed in the measured response. (**c**) Integrated data of the spectra illustrated in (**b**), each offset by 10 *pA* of current for clarity.

Here, *σ* is the width (standard deviation) of the underlying response in units of Tesla, Δ*I* is the change in current in Amps, and *B* is the magnetic field in Tesla where the current is maximum at *B* = 0*nT*. Magnetic field modulation will induce a series of harmonics in the measured response. The fundamental will have an envelope that is proportional to the responses derivative if the modulation amplitude *B*_*m*_ is relatively small. This concept is illustrated in Fig. 2a. The measured data resulting from demodulating the fundamental is,


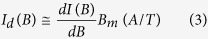


The uncertainty, or sensitivity, in the magnetic field *δB* is related to the uncertainty in the measured current response *δI*_*d*_ by,


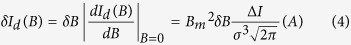


Additionally, the field modulation scheme allows for a measurement to be made that is shot noise limited (far from the flicker noise elbow). Therefore, the uncertainty in current response *δI*_*d*_ is related to shot noise by, 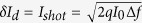, where *q* is the electronic charge, *I*_0_ is the DC current which is responsible for the flicker noise, and Δ*f* is the bandwidth of the measurement which is related to the time constant *τ* of the low pass filter that follows the mixer in the demodulator, Δ*f* *=* 1/2*πτ*. Setting eq. 4 equal to the shot noise and assuming *B*_*m*_ = *σ*, rearranging yields the sensitivity of the instrument,


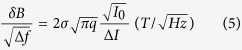


The same relation was used to calculate sensitivity limits by Baker *et al*. for an EDMR based magnetometer^36^. The change in current Δ*I* at *B* = 0 *mT* was extracted from the measured data by numerical integration. (Integration was performed *I*(*B*[*i*]) = *I*_*d*_(*B*[*i*])/(*F*_*s*_*B*_*m*_) + *I*(*B*[*i* − 1]) for *i* = 1:*M*, where *i* is the index of the array, *M* is the number of samples in the spectrum, and *F*_*s*_ is the sample rate in units of samples per unit magnetic field).

**Figure 3:**
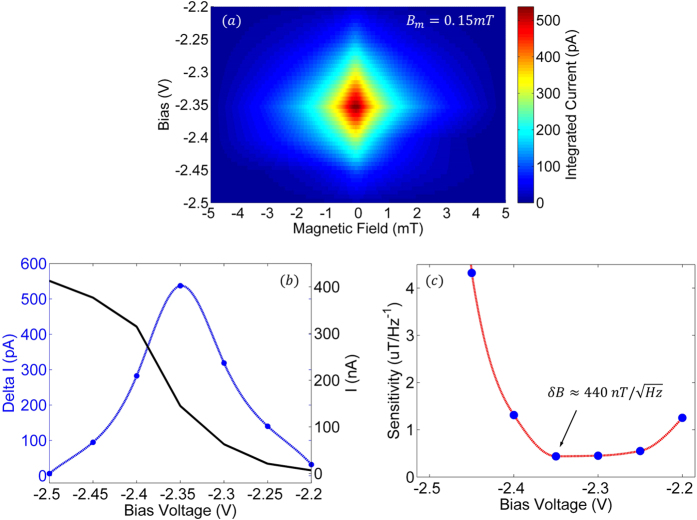
Panel (**a**) illustrates the change in current Δ*I* as a function of applied magnetic field and junction bias. The other two plots illustrate (**b**) the change in current Δ*I* at *B* = 0 *mT* and DC current *I*_0_ and (**c)** sensitivity *δB* as a function of applied junction bias. Note that for this particular SiC device, a forward bias of 2.35 *V* yielded the optimum sensitivity of 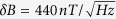.

Figure 2b illustrates the raw spectra acquired at various modulation amplitudes with a forward junction bias of 2.3 *V*. The peak-to-peak amplitude of the measured data grows with increasing modulation amplitude as it represents *dI*(*B*) rather than *dI*(*B*)/*dB*. The relative sharpness of the spectrum is attributed to the presence of a dominant defect, a silicon vacancy variant, within the ordered crystalline environment of the SiC semiconductor. The magnetoresistive response within the 4H-SiC lattice will therefore naturally have a sharper response (<0.3 *mT* in this case) than that observed in amorphous materials such as organic semiconductors and/or dielectric tunneling junctions^52^. As described earlier, the crystalline nature of the semiconductor allows for the detection of the electron-nuclear hyperfine interactions which are depicted by the inflections spaced 1.65 *mT* apart, symmetric about zero magnetic field. Additionally, as described earlier, the narrow notch located precisely at zero magnetic field (highlighted in the inset of the panel) is attributed to spin-spin interactions of the defect electron and can only be resolved when leveraging a low amplitude modulation waveform and an elevated junction bias. Its presence can therefore be turned on or off and leveraged for self-calibration which is described in more detail later in this report. Figure 2c illustrates the integrated responses of the spectra depicted in Fig. 3b, each offset by 10 *pA* for clarity. Note that, as expected, the change in current Δ*I* at *B* = 0 *mT* in the integrated spectra are all the same for different modulation amplitudes.

The junction bias was varied to find the optimal tradeoff between signal amplitude and device noise, and hence, sensitivity. Figure 3a illustrates a series of measurements made over a 1 *Hz (τ* = 1/2*π*) bandwidth at different junction biases. As illustrated in panels 3b and 3c, the maximum change in current (Δ*I/I* = 0.37%) was extracted from the integrated data to yield an approximate sensitivity of 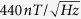 when a forward bias of 2.35 V was applied to the junction. Although the sensitivity reported here makes for a good magnetic field sensor on the surface of Earth (*B*_*Earth*_ ≈ 30,000 *nT*), its sensitivity is not ideal in its current state for planets with small magnetic fields. However, the sensor has great potential and a straightforward path toward substantial sensitivity improvement. The steps outlined to increase the sensitivity to a level competitive with state of the art planetary sensors are later described in detail.

## Vectorized Measurement

**Figure 4:**
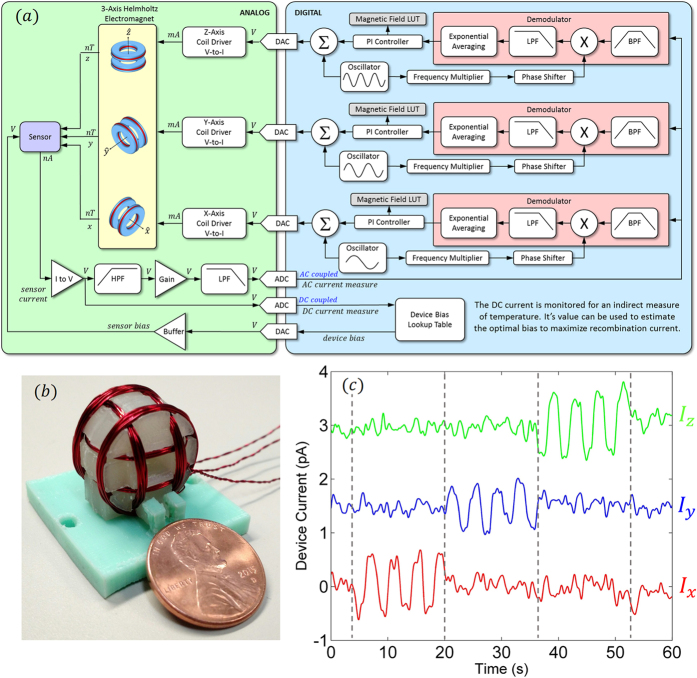
(**a**) Block diagram of the proposed magnetometer. (**b**) Photograph of our three-axis set of Helmholtz coils which houses the SiC sensor. (**c**) Measurement of three current components versus time, each offset by 1.5 *pA* for clarity, in the presence of an alternating axis, ±1500 *nT* square wave magnetic field.

The proposed magnetometer is based on a magnetic field cancellation scheme that maximizes the ZFSDR current in the pn junction by maintaining a local region of zero-magnetic field across the volume of the device. The device is housed within three sets of Helmholtz coils (one for each dimension) that are driven independently to provide a low-frequency (<10 Hz) cancellation field and a modulation field at audio frequencies. As the low-frequency driving current in these Helmholtz coils is directly proportional to the magnetic field it generates, its measure will serve as an indirect measure of the field being cancelled in each dimension. The block diagram of the magnetometer is illustrated in Fig. 4a and a picture of our 3D printed prototype of the coil system is illustrated in Fig. 4b.

The magnetic field induced change in SDR current of the SiC sensor is first fed through a high gain transimpedance amplifier with AC coupling before it is digitized for optimum sensitivity. (The amplified DC current will also be digitized in order to extract a crude measure of temperature. This will allow the instrument to optimally bias the SiC sensor for maximum recombination for any changes in temperature that may occur). The conditioned AC signal is then digitized and fed into three independent (one for each axis), user-configurable digital demodulators operating at different frequency bands. This allows for the three vectorized magnetic field components to be frequency division multiplexed onto a single channel. The demodulator involves an optional first stage of digital bandpass filtering, and then is followed by a mixer that performs a point-by-point multiplication of the incoming signal with a time-synchronized, user-configured (demodulating harmonic and demodulating phase) sinusoid. This operation allows one to measure the signal strength at any particular harmonic of the fundamental modulation frequency. A low-pass filter is used to remove the high-frequency mixing artifact, and an exponential averaging block is used to further reduce noise on the remaining signal based upon the user-configured bandwidth. A digital-based PI controller is implemented for each of the three demodulators for tracking the zero crossing center line of each current component. The error output of each controller is added to its corresponding modulation waveform in software prior to digital-to-analog conversion. This error is proportional to the magnetic field in each axis, and hence, is the current required to be passed through each Helmholtz coil in order to maintain a local region of zero magnetic field. In order to test our 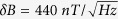 sensitivity metric, we recorded the current for each axis in the presence of an alternating axis, square wave magnetic field of amplitude of ±1500 *nT* within a mu-metal shielded chamber. As illustrated in Fig. 4c, the sensor can easily and simultaneously measure these weak magnetic field signatures using a single sensor.

## Self-calibration

**Figure 5:**
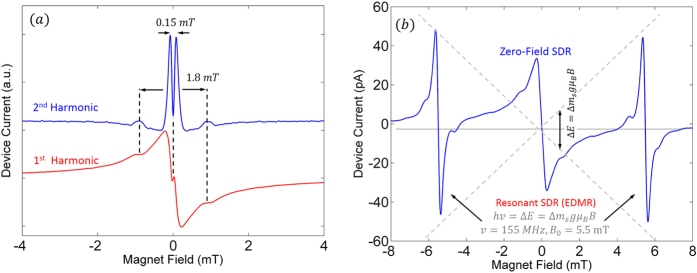
Self-calibration modes of SiCMag. (**a**) This figure illustrates that when the appropriate bias and modulation amplitude are selected, the magnetometer can operate in self-calibration mode. This bias dependent inflection at precisely zero magnetic field produces a sharp derivative feature in the second harmonic that is approximately 0.15 *mT* in spacing. As this feature is attributed to the strength of the spin interactions, the spacing will remain constant as a function of temperature and time. As a result, they are magnetic field markers that may serve to self-calibrate the magnetometer. (**b**) Alternative form of self-calibration leveraging EDMR. This method involves adding a small coil or resonator, built into the circuit board, which subjects the device to RF radiation. The field frequency relationship of the defect resonant transition (as well as the half-field forbidden transitions indicated by the red arrows) provides an absolute measurement which can be used to validate the ZFSDR measurement.

One of the biggest concerns with magnetometers, and scientific instruments on board satellites and landers in general, is remote calibration. SiCMag has the ability to self-calibrate, a significant advantage. SiCMag has two forms of self-calibration, both of which are illustrated in Fig. 5. The first and simpler self-calibration method entails leveraging the electron spin interactions that are observed in the ZFSDR spectrum. Figure 5a illustrates the 1^st^ and 2^nd^ harmonic spectra which clearly demonstrate the detection of the electron spin interactions discussed earlier. Because these interactions are virtually independent of temperature and also tremendously stable over long periods of time, their spacings will be maintained with extraordinary stability. (A previous work has demonstrated that the spacing of the narrow split peak observed in organic semiconductors remains constant from room temperature all the way down to 10 K^46^). By measuring the spacing of these magnetic field indicators as a function of the applied coil current, one is able to extract a constant-of-proportionality measure that can be used to calibrate the coil-driving system. This self-calibration mode can be turned on or off with the bias junction voltage and/or modulation amplitude as was shown earlier. The modulation amplitude essentially controls the minimum detectable feature size while the bias voltage not only controls sensitivity, but also controls the strength of the coupling between the two electrons spins. It should be noted that each axis will need to be calibrated independently to account for not only defect anisotropy but also due to differences in coil size and non-ideal characteristics of the electronics that drive each axis.

The second method of calibration entails performing low-field EDMR^35^,^36^,^37^,^52^ on the same device as illustrated in Fig. 5b. This method is appealing because the measurement itself becomes a form of absolute magnetometry, and can be used to validate the measured ZFSDR response. The method can be accommodated by incorporating a miniature RF excitation source which drives a small loop coil or strip line resonator on the PCB to provide the oscillating magnetic field required to drive the spin system into resonance. Although this adds a bit of complexity, the additional magnetic field measurement provides invaluable redundancy in the remote environment of space. It should be noted that this method shouldn’t be used when operating in a high energy radiation environment for prolonged periods of time. If the radiation induces a significant number of defects (with different g factors) due to prolonged exposure, the EPR condition of the defect electron outlined earlier may change. This adds uncertainty to the precise field/frequency relationship of the EPR condition, thereby making the measurement unreliable. However, the wide bandgap of the SiC semiconductor should ensure that measurement altering defects are not created, or at least not in an abundance that overwhelms the original defect spectrum. It should be noted that even if different defects are created in these harsh environments, it will not affect the magnetoresistive response associated with the device because the ZFSDR response is insensitive to defects with different g values. Thus, the accuracy of the measurement is preserved. However, the linewidth may be slightly compromised which results in a slight loss in sensitivity. Radiation tests will need to be performed in order to further quantify these cases.

## Future Work and Summary

**Figure 6:**
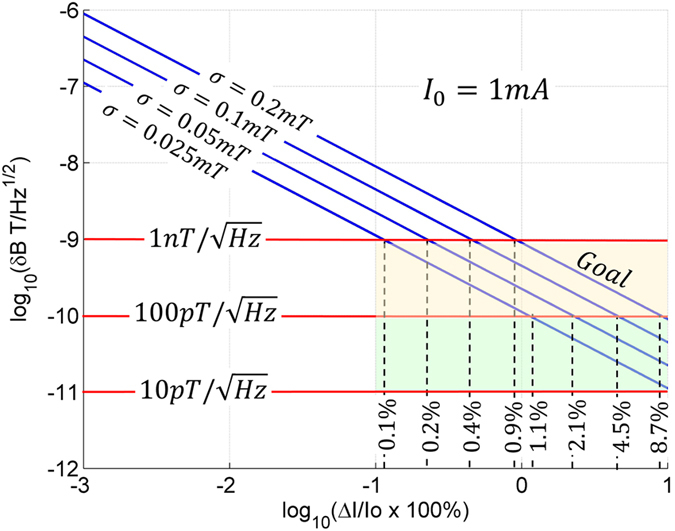
Theoretical sensitivity for a SiC sensor with *I*_0_ = 1 *mA*.

Although SiCMag, in its present form, is characterized as having a 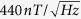 sensitivity, there are many ways to improve performance. First and foremost, we leveraged the pn junction within an experimental microelectronic device designed for high power applications, which was not optimized in any way for magnetometry. The next step in this work will involve developing a custom device designed to exploit the ZFSDR recombination phenomenon. Tradeoffs between geometry, sensing area, doping, and annealing will be made to find the optimum line shape and response. Once processed and fabricated, the sensitivity can be significantly enhanced by creating stable silicon vacancy defects by exposing the devices to high energy electron radiation as was done for optical magnetometry methods in SiC^40^,^41^,^42^,^43^. These defect engineering methods will help increase Δ*I* and improve sensitivity. Also, eventual device fabrication using isotopically pure SiC, that is SiC in which the crystal is depleted of ^29^Si or ^13^C, will sharpen the linewidth *σ*, which will push the sensitivity to its limits. Even though the hyperfine interactions are responsible for the magneto-response, we feel that the 1.1% magnetically abundant ^13^C and 4.7% magnetically abundant ^29^Si (and magnetic dopant atoms) contribute to the breadth of the signal and limit the ultimate sensitivity. Using an isotopically purer material will allows us to better control point defect creation and limit the amount of random electron nuclear hyperfine interactions, thereby sharpening the magneto-response. And finally, improved electronics which leverage low-noise ICs, layout practices, and sigma-delta ADC sampling should improve SNR, sensitivity, and stability of the measurement. Figure 6 illustrates the extrapolated sensitivity, given by eq. 5, as a function of percent change in current (100% *x*Δ*I*/*I*_0_) for devices with linewidths of *σ* = 0.2 *mT*, 0.1 *mT*, 0.05 *mT*, and 0.025 *mT* and *I*_0_ = 1 *mA* DC current for all four cases. As illustrated in the figure, the 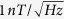 is easily attainable using the plan outlined above; however, it is still unclear if the sensitivity can be pushed into the 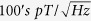 regime. We also plan on experimenting with diamond and GaN based semiconductor devices as they also have much promise in this application due to their relatively large bandgaps and crystalline structure. Diamond may eventually show to have the optimum response due to its sharp response attributed to the small number of hyperfine interactions from the ^13^C atoms.

The proposed magnetometer technology has enormous potential for the future of NASA and its space missions. As the magnetometer can be made extremely small, it has the potential to be manufactured into a microelectromechanical system (MEMS) device. Additionally, the magnetometer has the potential application to operate in high temperature and high radiation environments due to the wide bandgap and robustness of the SiC semiconductor. The magnetometer does not require any high frequency circuitry, thus reduces risk and eliminates the need to maintain strict temperatures for accurate and reliable magnetic field measurements. The combination of these features, along with being purely electrical and inexpensive, enables the technology to be used for a variety of magnetic field sensing applications, including planetary entry probes, landers, missions in extreme environments such as Venus and Jupiter, and in swarms of spacecraft significantly smaller than current nanosats.

## Methods

In this study, we use the source/drain to substrate pn junction of a 4H-SiC n-channel lateral MOSFET. The device was fabricated on a p-type substrate which was Al doped to about 6 × 10^15^. The source and drain n-wells were created by P implantation. The device has a channel area (L × W) of 400 × 400 *μm*^2^ and the gate oxide is a 50 *nm* thermally grown ONO stack (10 *nm* SiO_2_/30 *nm* Si_3_N_4_/10 *nm* SiO_2_) which received a 5 minute Ar anneal at 1600 °C.

We leveraged a custom designed preamp for amplification and signal conditioning. A NI USB-6289 M Series DAQ card was used to digitize the amplified device current and also used to generate three modulation waveforms with configurable offset, frequency, and phase. These signals were then fed into custom designed analog buffers which drove each of the three coils independently. LabVIEW software run on a Dell Inspiron laptop with 6 GB RAM, was used for signal processing and waveform generation. All measurements were made at room temperature. The low-field spectra were acquired on a custom built low-field EDMR spectrometer while the vectorized measurement illustrated in Fig. 4 were made in a mu-metal shielded chamber with three axis Helmholtz coil magnetic field generating source located at the Jet Propulsion Laboratory.

## Additional Information

**How to cite this article**: Cochrane, C. J. *et al*. Vectorized magnetometer for space applications using electrical readout of atomic scale defects in silicon carbide. *Sci. Rep.* **6**, 37077; doi: 10.1038/srep37077 (2016).

**Publisher's note:** Springer Nature remains neutral with regard to jurisdictional claims in published maps and institutional affiliations.

## Figures and Tables

**Table 1:** Hamiltonian matrix elements of singlet triplet pairs in the presence of a *I* = 1/2 nucleus.

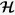	|*T*_+_, ↑〉	|*T*_0_, ↑〉	|*S*_0_, ↑〉	|*T*_−_, ↑〉	|*T*_+_, ↓〉	|*T*_0_, ↓〉	|*S*_0_, ↓〉	|*T*_−_, ↓〉
〈↑, ***T***_+_|	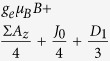	0	0	*D*_2_	0	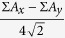	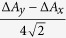	0
〈↑,***T***_0_|	0	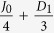	Δ*A*_*z*_/4	0	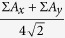	0	0	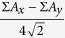
〈↑, ***S***_0_|	0	Δ*A*_*z*_/4	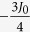	0	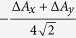	0	0	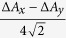
〈↑, ***T***_−_|	*D*_2_	0	0	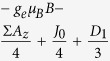	0	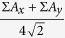	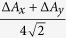	0
〈↓, ***T***_+_|	0	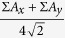	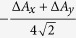	0	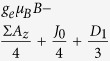	0	0	*D*2
〈↓, ***T***_0_|	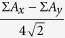	0	0	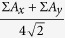	0	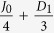	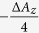	0
〈↓, ***S***_0_|	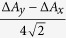	0	0	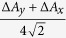	0	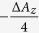	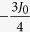	0
〈↓, ***T***_−_|	0	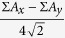	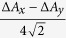	0	*D*_2_	0	0	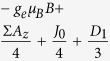

**Figure:**