# Erratum: Vectorized magnetometer for space applications using electrical readout of atomic scale defects in silicon carbide

**DOI:** 10.1038/srep40834

**Published:** 2017-01-16

**Authors:** Corey J. Cochrane, Jordana Blacksberg, Mark A. Anders, Patrick M. Lenahan

Scientific Reports 6: Article number: 37077; 10.1038/srep37077 published online: 11 28 2016 updated: 01 16 2017.

This Article contains a typographical error. In the Results section, under the subheading “SiC Sensor”

“These intermediate coupled pair states can be described by the singlet-triplet basis: the symmetric triplet states, T+ = |↑↑〉, T0 = (|↑↓〉 + |↓↑〉)/, and T− = |↓↓〉, each having spin angular momentum S = 1 with ms = +1, 0, −1 respectively or the anti-symmetric singlet state, S0 = (|↑↓〉 + |↓↑〉)/ which has spin angular momentum S = 0 with ms = 0”.

should read:

“These intermediate coupled pair states can be described by the singlet-triplet basis: the symmetric triplet states, T+ = |↑↑〉, T0 = (|↑↓〉 + |↓↑〉)/, and T− = |↓↓〉, each having spin angular momentum S = 1 with ms = +1, 0, −1 respectively or the anti-symmetric singlet state, S0 = (|↑↓〉 − |↓↑〉)/ which has spin angular momentum S = 0 with ms = 0”.

